# Epigenetics Plays a Role in the Pathogenesis and Treatment of Diabetes

**DOI:** 10.3390/genes16070769

**Published:** 2025-06-30

**Authors:** Kajetan Kiełbowski, Estera Bakinowska, Andrzej Pawlik

**Affiliations:** Department of Physiology, Pomeranian Medical University, 70-111 Szczecin, Poland; kajetan.kielbowski@onet.pl (K.K.); esterabakinowska@gmail.com (E.B.)

**Keywords:** diabetes, epigenetics, DNA methylation, histone modifications

## Abstract

Diabetes is a chronic and progressive metabolic disease that is associated with increased blood glucose levels. Recently, accumulating evidence has demonstrated that the pathophysiology of the disease involves changes within the epigenome. Epigenetics studies the role of DNA methylation, histone modifications, and non-coding RNA in gene expression regulation. Aberrant epigenetics changes the profile of gene expression and is strongly associated with the pathophysiology of various diseases. In the context of diabetes, hyperglycemia affects the profile of DNA methylation and histone modifications, which can be used as biomarkers or therapeutic targets. The aim of this review is to discuss the latest findings linking epigenetics and diabetes.

## 1. Introduction

### 1.1. Introduction to Diabetes

Diabetes mellitus (DM) is a group of chronic metabolic disorders characterized by elevated blood glucose levels due to inadequate insulin action. The pathophysiology of the condition greatly depends on the subtype of diabetes. Type 1 DM (T1DM) results from the autoimmune destruction of pancreatic β-cells. In contrast, type 2 DM (T2DM) is associated with insulin resistance and hyperinsulinemia, a highly complex condition involving several organs and tissues [1]. DM refers to other subtypes of the condition as well, such as monogenic diabetes and gestational DM (GDM) [2]. Different treatment options are used depending on the subtype of DM. While insulin remains a primary drug in T1DM, several other options are available for patients with T2DM. For instance, metformin, GLP-1 analogs, SGLT-2 inhibitors, and DDP-4 inhibitors can be used in patients with T2DM [3]. Understanding the processes that drive the progression of DM allows for the introduction of novel treatment strategies and improved clinical benefits. For instance, knowledge of mechanisms induced by GLP-1, such as increased insulin secretion, reduced glucagon release, and inhibited gastric emptying, resulted in the introduction of GLP-1 analogs and a series of investigations targeted at creating novel molecules [4].

### 1.2. Introduction to Epigenetics

**Figure 1:**
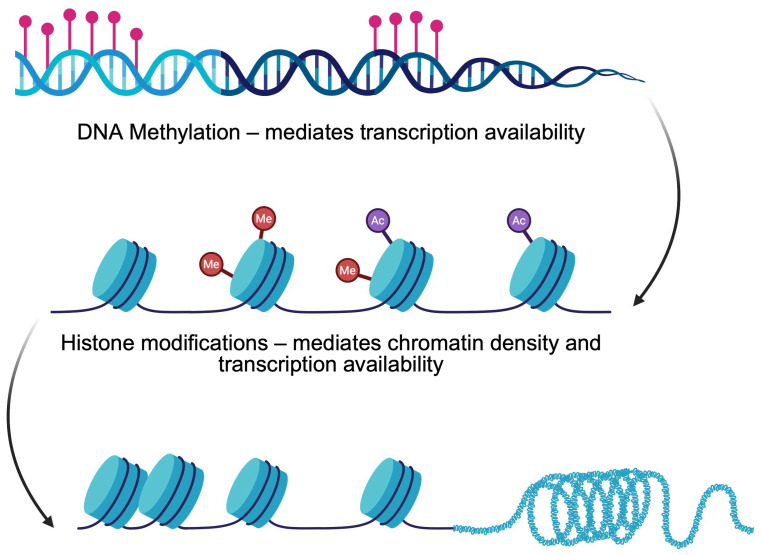
Classic epigenetic mechanisms regulating gene expression. Created in BioRender. Physiology, D. (2025) https://BioRender.com/8bgsr37 (accessed on 20 May 2025).

Currently, numerous studies aim to investigate pathophysiology pathways to identify potential therapeutic targets. Recent findings highlight the role of epigenetic mechanisms—heritable modifications of gene expression without changes in DNA sequence—in the pathogenesis of DM. Accumulating evidence demonstrates that epigenetics can either be used as biomarkers or in therapeutic processes. Key processes include DNA methylation and histone modifications that regulate the process of transcription and influence β-cell development, insulin secretion, and disease progression (Figure 1). Furthermore, the activity of non-coding RNA in gene expression regulation is considered one of the key elements in mediating gene expression. Alterations in epigenetics create imbalances that eventually lead to the pathogenesis of diseases. It is essentially important in diabetes as anti-diabetic drugs, such as GLP-1 analogs, can revert epigenetic alterations induced by high glucose [5]. This review aims to summarize the current evidence on how DNA methylation and histone modifications contribute to the onset and progression of DM, focusing on β-cells physiology and therapeutic implications.

### 1.3. Methodology

To identify studies relevant for this narrative review, the following search terms were used in the PubMed database: ‘diabetes’, ‘epigenetics’, ‘DNA methylation’, ‘histone modifications’, ‘histone methylation’, ‘histone acetylation’, ‘histone lactylation’, and their combinations. In general, studies published within the last 5 years were included in the discussion. Older articles were cited only if there was a broader context required when a new term was being introduced.

## 2. Epigenetics and Diabetes

### 2.1. DNA Methylation

DNA methylation is one of the basic mechanisms of epigenetics that mediates gene expression. Classically, hypermethylation of gene promoters is associated with suppressed transcription. By contrast, hypomethylation promotes gene transcription. DNA methylation is strongly involved in healthy and pathological states. DNA methylome changes during embryonic development, thus contributing to the regulation of cell differentiation. Changes in methylation can be observed in ageing cells as well as in pathologies with complex pathogenesis, such as malignancies [6]. Furthermore, DNA methylation can represent a link between risk factors and the development of a particular condition. Changes in DNA methylation profiles have been investigated as prediction biomarkers to analyze health outcomes; the majority of studies are associated with the field of oncology [7]. However, novel findings were published regarding the role of DNA methylation in diabetes [8,9,10].

To begin with, DNA methylation influences the activity and maturity of the pancreatic β-cells. Specifically, β-cell maturation increases methylation of tyrosine hydroxylase (TH), an enzyme catalyzing the synthesis of catecholamines, which is inversely associated with insulin secretion. Ablation of methyltransferase in endocrine progenitors significantly increases the abundance of TH-positive β-cells [11]. Hypothetically, increased TH promoter methylation could affect the efficacy of the pancreas to secrete insulin. Recently, researchers studied preclinical mouse models fed with a high-fat and high-sodium chloride diet. They observed that dapagliflozin promoted insulin secretion and reduced TH-positive areas within the Langerhans islets [12]. Therefore, pancreatic TH could represent a promising therapeutic target. Moreover, findings add further evidence on the beneficial role of dapagliflozin, an SGLT2 inhibitor that suppresses the reabsorption of glucose from primary urine. It is one of the primary drugs recommended for patients with T2DM [3]. Adenosine kinase (ADK), which phosphorylates adenosine to AMP, is also related to DNA methylation and pancreatic development. Overexpression of ADK impairs pancreatic functionality by promoting glucose intolerance, reducing β-cell mass and fasting insulin plasma levels, while β-cells proliferation downregulates ADK. Epigenetics seems to link ADK with pancreatic cells, specifically, the knockdown of DNMT3A reversed the suppression of β-cells proliferation induced by ADK overexpression [13]. Thus, DNA methylation is among the important processes driving the physiological composition and functioning of β-cells.

Moreover, the pathogenesis of diabetes also involves processes related to an altered DNA methylome. Firstly, the state of hyperglycemia impacts DNA methylation profile in embryonic stem cells, including differentiation towards pancreatic lineages [14]. These findings suggest a mechanistic link between GDM in mothers and the risk of developing diabetes later in life by their offspring [15]. Adding to this evidence, GDM in mice is associated with oocyte DNA methylation changes in offspring, which could explain the risk of developing metabolic conditions [16]. Such observations also spark a discussion of whether hyperglycemia can affect pancreatic cell development later in life. Although it is debatable whether pancreatic progenitor cells exist [17], current evidence demonstrates that they are not a source of new β-cells. In contrast, under extreme situations, such as the ablation of most of β-cells, non-β-cells were found to promote the development of new insulin-positive cells [18]. Further experiments demonstrated that the dedifferentiation/proliferation/redifferentiation pathway is involved in the regeneration of β-cells after ablation. Importantly, surgical removal of β-cell mass also promoted β-cells regeneration [19]. Furthermore, other metabolic conditions such as dyslipidemia could also affect the above-mentioned mechanisms. Nevertheless, the following assumptions could be made. Hyperglycemia can disrupt the processes related to pancreatic cell development. The latter process depends on DNA methylation as its modifications can alter pancreatic proliferation. It is an open question if and how DNA methylation can promote the process of β-cell differentiation in an adult.

These discussions involve mechanisms of pancreatic development or regeneration of β-cells. Nevertheless, abnormal glucose levels or the state of diabetes impact β-cells themselves. In an in vitro experiment using β-cells, high concentrations of glucose significantly affected the DNA methylome. Using a genome-wide methylation analysis, Alhazzaa et al. [20], detected 478 differentially methylated gene promoters in human pancreatic β-cells derived from induced pluripotent stem cells. Researchers performed functional analyses, which demonstrated alteration of several major pathways, including semaphorin cascade, hypoxia in the cardiovascular system, and nNOS signaling in skeletal muscle cells, among others [20]. These experiments offer an insight into the relationship between epigenetics and the pathophysiology of diabetes. Simultaneously, treatment of diabetes is suggested to alter DNA methylation within the β-cells. In mouse models, the use of dapagliflozin was revealed to significantly affect DNA methylation in the islets. These epigenetic alterations lead to changes in gene expression, thus having a profound effect on pancreatic cells [8].

**Figure 2:**
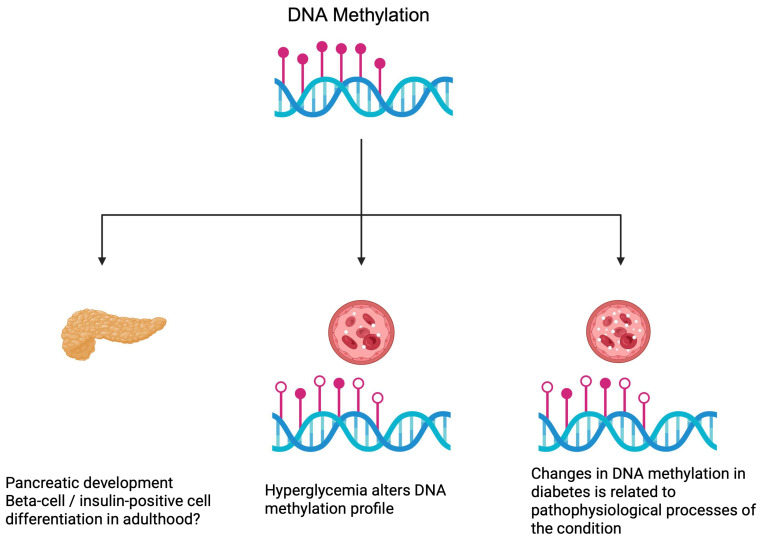
DNA methylation contributes to pancreatic development and pathophysiological mechanisms of diabetes. Created in BioRender. Physiology, D. (2025) https://BioRender.com/5iok4v8 (accessed on 20 May 2025).

DNA methylation profile is altered in patients with diabetes and diabetic complications [21,22,23], which is in line with the preclinical data. These abnormalities create opportunities to study diabetes pathogenesis from a different perspective and identify novel biomarkers of the disease. Recently, a more direct association between methylation patterns and T2DM was discovered. Lai et al. [9] demonstrated that methylation changes over time, with significant differences observed between patients with T2DM and people with normal glucose tolerance. Furthermore, the authors noticed alterations in methylation associated with the status of diabetes. Seven CpG sites have relationships with the progression from normal glucose tolerance to prediabetic conditions or to diabetes. Intriguingly, monitoring of DNA methylation has been suggested to be used in the identification of diabetic cases years before a diagnosis can be established [23]. Hypothetically, further improvement in this area could lead to the selection of a cohort with a greater risk of developing diabetes. Perhaps, appropriate lifestyle modification or pharmacological treatment in selected cases could prevent full-scale diabetes from occurring. Additionally, DNA methylation could represent causative factors of diabetes [24]. In 2024, Carry and colleagues [25] analyzed DNA methylation in patients with islet autoimmunity, a process preceding the development of T1DM. The authors observed that methylation changes in patients who lose autoantibodies, as compared to patients with continuous presence of antibodies and to those who progress. Additionally, methylation monitoring can provide beneficial information regarding complications of diabetes. DNA methylation of the *KIF16B* gene is associated with the incidence of proliferative retinopathy in patients with T1DM. Specifically, increased methylation correlated with reduced risk of the condition developing. The gene encodes a protein involved in preventing iron accumulation within the retina, which is related to oxidative stress and promotes retinopathy [10]. These findings could offer a possibility to use epigenetics to monitor the potential development of the disease and its complications. Furthermore, such studies suggest potential therapeutic targets that could be used to prevent or treat diabetes. Figure 2 demonstrates the importance of DNA methylation under physiological and pathological conditions.

The potential role of methylation of individual gene regions has shed more light on the pathogenesis mechanism in this metabolic condition. Methylation of the cg19693031 site within the 3′-untranslated region on the thioredoxin-interacting protein (*TXNIP*) gene has been associated with fasting glucose levels and HbA1c values [26]. Furthermore, hypomethylation of TXNIP-cg19693031 is observed in patients with T2DM [27]. Moreover, reduced methylation levels of this region were linked with poor long-term glycemic control in patients with T1DM [28]. Thus, classic mechanics explaining DNA methylation activity demonstrate that a higher expression of TXNIP could be related to diabetes pathogenesis. In line with this hypothesis, the expression of TXNIP has been correlated with the pathophysiology of diabetes. In mice with streptozotocin-induced conditions, overexpression of TXNIP reduced the weight mass of pancreatic beta cells, thus increasing the severity of the disorder [29].

The presence of diabetes is associated with non-alcoholic fatty liver disease (NAFLD) [30]. TXNIP was also found to link T2DM and NAFLD occurrence and severity [31]. Moreover, TXNIP has been correlated with typical complications of diabetes, such as diabetic nephropathy or vascular complications [32,33]. It has been suggested as a potential target in the treatment of diabetes, with researchers trying to develop inhibitors of the protein [34]. Epigenetic control of TXNIP expression seems to be one of the mechanisms that could be targeted and regulated to affect the severity of diabetes. Current methods of epigenetic regulation are non-selective and mostly studied in cancer models. However, in 2016, Vojta et al. [35] described a method implementing the CRISPR-Cas9 system to induce targeted methylation. Intriguingly, the system was recently described to reduce pathological features of Alzheimer’s disease in vivo. These findings are promising regarding targeted change of DNA methylome to selectively alter gene expression [36].

Recently, more data has been shared regarding the epigenetics of the *KCNJ11* gene. It encodes a subunit of the potassium channel that plays a role in insulin secretion. Zhu et al. [37] demonstrated that patients with a new onset of T2DM have lower methylation levels of the *KCNJ11* promoter. In the case of the *KCNJ11*, genetics is important as well. Mutations of the gene are associated with neonatal and MODY diabetes [38,39].

An interesting approach to T2DM monitoring has been recently suggested. Liquid biopsy is a procedure highly investigated in the field of oncology. It allows for the study of tumor-free DNA molecules and the identification of potential mutations and therapeutic targets. Karaglani and colleagues [40] demonstrated that liquid biopsy can be performed in patients with diabetes. However, as the diagnosis of diabetes itself is a relatively straightforward procedure, liquid biopsy could be implemented to monitor disease development in patients prone to diabetes or to monitor treatment response. The authors studied circulating cell-free DNA molecules and analyzed their potential sources and methylation. Researchers demonstrated significant differences in the methylation profile of several genes between patients with T2DM and healthy individuals.

### 2.2. Histone Modifications

#### 2.2.1. Histone Methylation

Along with DNA methylation, histone modifications represent a second key epigenetic mechanism mediating gene transcription. Furthermore, there is a relationship between DNA methylation and histone modifications, as the latter mechanisms can influence the activity of the former [41]. This association creates an additional layer of regulation, which makes the network of epigenetics more complex. Histone modifications involve methylation, acetylation, ubiquitination, and lactylation, among others. These mechanisms change the accessibility of chromatin to processes mediating gene expression. Some modifications are classically considered as promoting or suppressing transcription. In addition, specific modifications can affect the occurrence of other modifications, which makes the interaction network more complex [42]. Decades of epigenetic research have demonstrated the importance of histone modification dysregulation in the pathogenesis of diseases. Similarly, these mechanisms participate in metabolic abnormalities associated with diabetes and the pathogenesis of the disease and its complications. Histone lactylation is a relatively novel modification that is strongly linked to metabolism. The primary substrate for this modification is lactate, which is generated from glycolysis and glutamine metabolism. Despite its recent identification, it is known that the process is involved in embryogenesis, metabolic reprogramming, wound healing, cellular differentiation, and regulation of immune responses [43].

**Figure 3:**
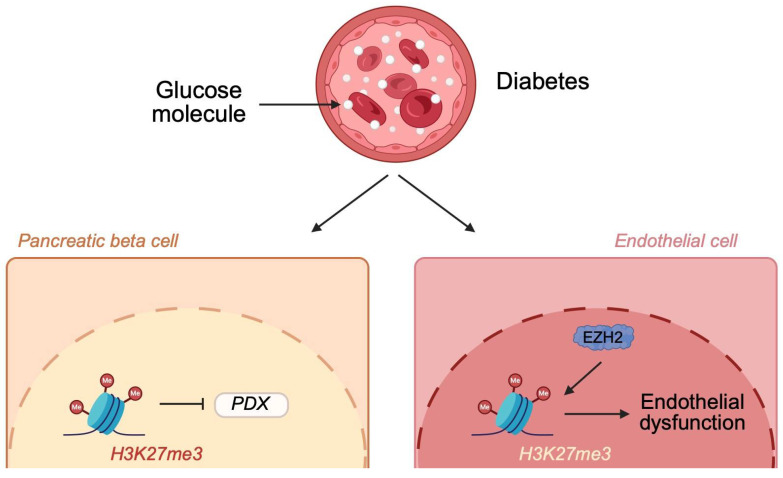
Higher glucose concentrations present in diabetes change the methylation profile, which affects the pathophysiology of diabetes. Specifically, it promotes the expression of H3K27me3, which is associated with dysfunction of pancreatic beta cells and endothelial dysfunction. Created in BioRender. Physiology, D. (2025) https://BioRender.com/zmk8oa4 (accessed on 20 May 2025).

Histone methylation processes are catalyzed by histone methyltransferases. Methylation frequently occurs at lysine and arginine residues. Classically, H3K4me3 and H3K27me3 are associated with the promotion and suppression of transcription, respectively. H3K9 methylation is considered repressive as well [44]. Recently, investigations concerning histone methylation shed new light on the pathogenesis of diabetes. In mice with streptozotocin-induced diabetes, an increased methylation of H3K9 (H3K9me2) within the pancreas was observed. Mechanistically, increased methylation was related to reduced expression of *Pdx*, one of the crucial transcription factors responsible for β-cell development. Treatment with exenatide (GLP-1 agonist) restored methylation levels of H3K9 [45]. Thus, such findings suggest that diabetes affects the epigenome of the pancreas. Consequently, pancreatic cells have reduced ability to produce insulin. Moreover, treatment of diabetes itself can reverse epigenetic alterations. Perhaps, we could target enzymes responsible for histone modifications to further increase pancreatic functionality in the future. High glucose concentrations affect other cells as well. In aortic endothelial cells exposed to high glucose, an increased abundance of H3K27me3 was observed. The observed changes were accompanied by increased expression of the enhancer of zeste homolog 2 (EZH2), a histone methyltransferase. High glucose levels are related to endothelial dysfunction, a major mechanism driving vascular pathologies. Suppression of EZH2 expression could reverse inflammatory responses observed in the endothelium [46] (Figure 3). Therefore, these findings demonstrate that abnormal glucose concentrations induce epigenetic modifications that change gene expression and promote the pathogenesis of vascular complications frequently observed in patients with diabetes. EZH2 is also involved in pathologic alterations of the cardiac tissue in animals under hyperglycemic conditions [47]. These observations point to EZH2 as a potential therapeutic target that could be utilized in the treatment of diabetes. Al-Hasani et al. [48] showed that inhibition of EZH2 could restore pancreatic insulin secretion and represent a replacement method. Diabetes is also associated with the development of periodontal disease. Current evidence suggests there is an involvement of histone methylation in the pathophysiology of this condition. Specifically, diabetes in rats upregulates H3K4me3 and SETD1A in gingival tissues. Consequently, it increases the expression of matrix metalloproteinases 1 and 13 (MMP1 and MMP13). Suppression of the enzyme involved in histone methylation counteracts these results and downregulates MMP1 and MMP13 enzymes [49].

Diabetes also affects the epigenetics of Ras-related C3 botulinum toxin substrate 1 (Rac1). It is a cytosolic component of NOX2, one of the subunits of NADPH. Kowluru et al. [50] demonstrated that diabetes is associated with the greater presence of H3K9me3 at the Rac1 promoter. Simultaneously, researchers detected an upregulation of Suv39H1, a histone trimethyl-transferase. Suv39H1 silencing reduced Rac1 expression in in vivo experiments. The Rac1-NOX2 activation is related to the production of reactive oxygen species (ROS) and to the pathogenesis of diabetic retinopathy [51]. As a chronic metabolic condition with implications throughout the organism, studies are exploring potential links between DM and osteoporosis. Although the results are controversial, recent reports described the involvement of histone methylation in bone marrow mesenchymal cell differentiation to osteoblasts under diabetic conditions. Experiments demonstrated that diabetes reduces osteoblastic differentiation potential, downregulates KDM6B demethylase, and upregulates EZH2. Furthermore, an increased presence of H3K27Me3 was identified at the promoter of the BMP4 gene, which is a crucial factor in the osteoblastic differentiation process [52].

#### 2.2.2. Histone Acetylation

Histone acetylation is the other mechanism belonging to the histone modifications. Acetylation occurs at the N-terminal region or the internal lysine. Key enzymes that mediate these processes are histone acetyltransferases (HATs) and histone deacetylases (HDACs). The latter group involves NAD+-dependent sirtuins, a crucial family of enzymes highly investigated for their immunoregulatory properties. Acetylation is associated with major cellular biological processes, immune responses, neurological signaling, and ageing, while its abnormalities have been detected in metabolic conditions and cancer [53].

Cellular experiments demonstrated that high glucose concentrations affect histone acetylation. For example, these conditions promote acetylation of H3K18 and H3K27 [54] that mediates gene expression. However, further studies are required to confirm whether these effects participate in diabetes pathogenesis or if they are compensatory mechanisms. Moreover, Shrestha et al. [55] studied the effects of neutrophil glucose stimulation. The researchers observed that cells isolated from patients with diabetes more actively secrete neutrophil extracellular traps (NETs). These structures are composed of granules and double-stranded DNA molecules that participate in inflammatory responses. Additionally, the authors revealed that high glucose concentrations lead to the accumulation of acetyl-CoA, a major acetyl source of histone acetylation. Interestingly, an inhibitor of histone acetyltransferase suppressed the priming of NETs. NETs are considered to participate in the pathogenesis of typical diabetic complications, which was elegantly reviewed in an article by Shafqat and colleagues [56]. Therefore, abnormal histone acetylation does promote pathological responses observed in patients with diabetes.

**Figure 4:**
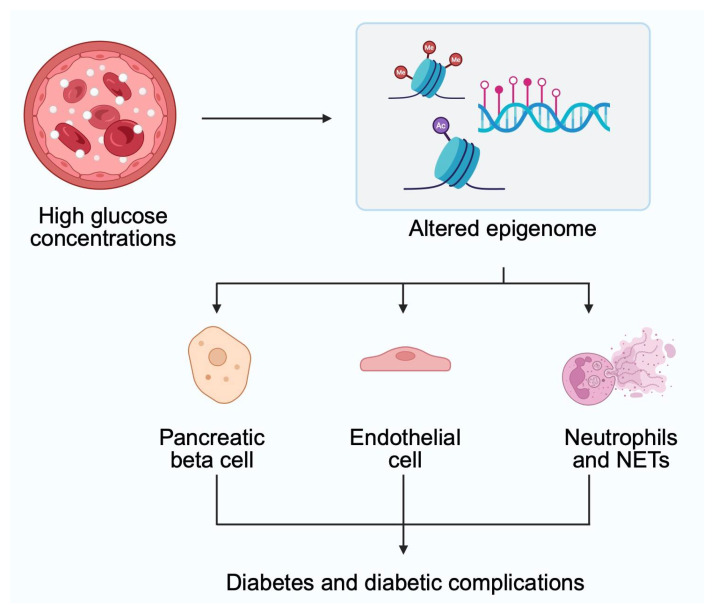
High glucose conditions change the epigenome of several types of cells, which broadly influences the pathogenesis of diabetes and its typical complications. Created in BioRender. Physiology, D. (2025) https://BioRender.com/jw7fzcv (accessed on 20 May 2025).

The previously mentioned study highlights the important process involved in the pathophysiology of diabetes complications. Namely, it is the accumulation of acetyl-CoA, a major element in metabolic pathways. Several studies demonstrated the importance of acetyl-CoA in the histone acetylation process [57,58,59]. Therefore, it indirectly influences chromatin accessibility, thus regulating gene expression and DNA repair. Conditions associated with the accumulation of acetyl-CoA are then more prone to alterations in epigenetics. Recently, researchers demonstrated that acetyl-CoA is involved in the pathogenesis of renal complications of diabetes [60,61]. The impact of acetyl-CoA on histone acetylation is one of the mechanisms linking histone modifications and diabetic kidney disease [60] (Figure 4).

Studies that examine the involvement of enzymes catalyzing histone acetylation or deacetylation add more evidence on the role of histone acetylation in diabetes. In addition, such investigations offer insight into potential therapeutic targets that could be used in the treatment of diabetes in the future. For example, lysine acetyltransferase 2 A (Kat2a) is one of the enzymes catalyzing histone acetylation. By increasing H3K27ac, the enzyme stimulates ferroptosis and is considered a participant in the pathogenesis of diabetic cardiomyopathy. Kat2a deficiency suppressed the condition in animal experiments [62]. Interestingly, the Kat2a-specific inhibitor has been previously examined in the context of arthritis, where it suppressed inflammatory responses [63]. This observation is promising for future development regarding inflammatory responses observed in patients with diabetes. Similarly, targeting acetyltransferase could provide beneficial outcomes in patients with diabetic kidney disease. Inhibition of p300 prevents the development of pathophysiological changes of diabetic kidney disease (e.g., glomerular hypertrophy) in animal models. Furthermore, it downregulates the expression of pro-fibrotic and pro-inflammatory molecules, which are strongly linked pathology of the kidneys [64]. The activity of p300 was also related to the occurrence of muscle atrophy present in T2DM. Through the suppression of p300 expression, it is possible to regulate autophagic flux and reduce muscle wasting [65].

Other enzymes involved in histone acetylation and diabetes are HDACs. Over the years, the inhibition of HDACs has been studied in the context of various conditions, such as cancer [66], pulmonary hypertension [67], and inflammatory and fibrotic diseases [68], among others. In diabetes, early experiments demonstrate potentially promising results. In a study utilizing diet-induced obese mice, an application of HDAC6 inhibitor was associated with weight loss and improved leptin signaling. Leptin resistance is one of the key features driving the pathogenesis of obesity and obesity-related inflammatory disorders. However, the authors demonstrated that the blood–brain barrier permeable HDAC6 inhibitor improved glucose tolerance as well [69]. As the pathogenesis of diabetes and obesity are interconnected, and leptin resistance also plays a role in pathological processes observed in diabetes [70], these findings are reasonable. In addition, the activity of HDAC was correlated with diabetic complications. For example, tissue sample analyses of patients with diabetic nephropathy confirmed an increased expression of HDAC6 compared to patients with minimal change disease. Further animal experiments showed that the use of the HDAC6 inhibitor reduced pathological renal changes. Mechanistically, the agent was found to suppress pyroptosis mediated by the NOD-like receptor (NLR) family pyrin domain-containing 3 (NLRP3) inflammasome [71]. These are important findings as NLRP3 is an important pro-inflammatory mediator, the activity of which is implicated in the progression of T2DM and its typical complications (nicely summarized in a review by Li and colleagues [72]). Interestingly, HDACs also contribute to the pathophysiology of T1DM. Recently, an interesting mechanism was described by Hu and collaborators [73]. Researchers studied the role of HDAC3 and lymphocyte activity in the context of T1DM. The authors demonstrated that HDAC3 regulates the expression of miR-296-5p, a member of the microRNA family. As a result of this interaction, apoptosis of lymphocytes was suppressed. However, these findings highlight relationships between different epigenetic mechanisms, thus demonstrating a broad network of interactions that make gene expression regulation more complex. Bakhdar et al. shed more light on the present discussion. In their report, the authors demonstrated that the use of vorinostat, an FDA-approved HDAC inhibitor, can suppress the development of diabetes induced by tacrolimus in in vivo experiments [74]. Despite vorinostat being approved for the treatment of cutaneous T cell lymphoma, such findings could pave the way for additional indications for well-known drugs.

#### 2.2.3. Histone Lactylation

Histone lactylation was described in 2019 [43]. However, despite its relatively recent identification, current evidence demonstrates the involvement of this histone modification in the pathogenesis of diabetic complications. For example, in diabetic kidney disease, renal lactic acid levels are positively correlated with serum creatinine, blood urea nitrogen, and urine albumin levels, as well as with the expression of several pro-fibrotic genes in mouse models of diabetes. Accordingly, the condition is also linked with increased histone lactylation (H3K27la, H3K23la, H3K9la, H3K14la, and H3K18la). Similarly to other histone modifications, lactylation does affect gene expression. For instance, H3K14la contributes to *klf5* upregulation, targeting of which suppresses epithelial-to-mesenchymal transition within diabetic kidneys [75]. In another in vivo experiment, therapies lowering plasma lactate levels improved renal parameters in diabetic models [76]. Another condition in which histone lactylation is possibly involved in diabetes-related cognitive impairment. The precise pathophysiology of this complication is not known, and the mechanisms involved differ depending on the type of diabetes. However, disrupted insulin signaling and cerebral lesions are being observed in patients with T2DM, which is thought to link the occurrence of metabolic disease with cognitive abnormalities [77]. Regarding T2DM, the levels of lactate pan histone lysine lactylation are increased in animal models. Through the H3K12la/FOXO1 pathway, lactylation can promote mitochondrial damage and contribute to cognitive disorders [78].

## 3. Limitations

Epigenetics has shown its involvement in the pathophysiology of numerous diseases. Accordingly, many epigenetic molecules have been suggested as potential diagnostic or therapeutic targets. Nevertheless, it should be noted that many of the promising results discussed here were achieved in in vitro and in vivo experiments. It is widely known that translation of preclinical successes to the clinic is a long-term and costly process filled with challenges. For example, it is estimated that approximately 90% drug candidates will not demonstrate clinical benefit in clinical studies [79]. The human organism is a highly complex structure with large networks of regulatory molecules that create a vastly different environment than cellular or animal models. Although the initial studies are promising and require further research, critical reflection is still needed when evaluating these early steps. However, if scientific determination lasts, novel breakthroughs are possible. For instance, epigenetics is a field which found clinical implications that are used in the clinic. MGMT promoter methylation is an important biomarker in the management of glioblastoma. Furthermore, HDAC inhibitors are registered in the treatment of cancer [66]. Thus, there are potential targets, but their clinical application is not yet assured. These early results should be validated and used in the design of further and novel experiments.

## 4. Conclusions

To conclude, the main take-home message from the recently published studies is that epigenetics plays an important role in the pathogenesis of diabetes. High glucose concentrations alter the epigenome, including DNA methylation and histone modifications, and this strongly affects gene expression. Consequently, these abnormalities participate in the progression of diabetes and its typical complications. Altered epigenetic mechanisms are observed in embryonic cells, lymphocytes, pancreatic cells, as well as in endothelial cells, renal cells, cardiomyocytes, and neutrophils, among others. These findings suggest that epigenetics is involved in GDM, T1DM, T2DM, diabetic nephropathy, cardiomyopathy, retinopathy, and wound healing. These observations could be translated into clinical practice. For instance, monitoring of the state of the epigenome could be used as a diagnostic biomarker. Furthermore, such analyses could be utilized to monitor treatment response. The development of gene panels to study their methylation may provide a platform to analyze the risks of diabetes progression and the occurrence of typical complications. In addition, understanding epigenetic mechanisms should be further studied to identify novel therapeutic targets or to use combination strategies that involve drugs that modify epigenetics.

## Data Availability

Not applicable.

## Abbreviations

The following abbreviations are used in this manuscript:

DM	Diabetes mellitus
T1DM	Type 1 diabetes mellitus
T2DM	Type 2 diabetes mellitus
ADK	Adenosine kinase
TH	Tyrosine hydroxylase
TXNIP	Thioredoxin-interacting protein
NAFLD	Non-alcoholic fatty liver disease
EZH2	Enhancer of zeste homolog 2
Rac1	Ras-related C3 botulinum toxin substrate 1
HATs	Histone acetyltransferases
HDAC	Histone deacetylase
NLRP3	NOD-like receptor (NLR) family pyrin domain-containing 3
